# Lowering the burden: Shorter versions of the Program Sustainability Assessment Tool (PSAT) and Clinical Sustainability Assessment Tool (CSAT)

**DOI:** 10.1186/s43058-024-00656-y

**Published:** 2024-10-10

**Authors:** Sara Malone, Kim Prewitt, Virginia McKay, Luke Zabotka, Caren Bacon, Douglas A. Luke

**Affiliations:** 1 Division of Public Health Sciences, Washington University School of Medicine, 600 S Taylor Ave, Saint Louis, MO 63110 USA; 2 Center for Public Health Systems Science, Washington University in St. Louis, St. Louis, MO USA

**Keywords:** Measurement development, Psychometrics, Program sustainability, Clinical sustainability, Organizational capacity

## Abstract

**Background:**

Although significant advances have been made in the conceptualization of sustainability, having pragmatic, psychometrically valid tools remains a need within the field. Our previous work has developed frameworks and tools to assess both program sustainability and clinical sustainability capacity. This work presents new, psychometrically tested short versions of the Program Sustainability Assessment Tool (PSAT) and the Clinical Sustainability Assessment Tool (CSAT).

**Methods:**

These methods were conducted in identical, parallel processes for the CSAT and PSAT. Previously collected data for these instruments was obtained across a variety of settings, contexts, and participants. We first conducted testing to determine cronbach’s alpha of shortened domains (3 items each) and then conducted Confirmatory Factor Analysis to ensure that the domains were still appropriate for the tool. After, the team met to review the results and determine the final versions of the short PSAT and short CSAT.

**Results:**

The short PSAT retained cronbach’s alpha’s of 0.82 – 0.91 for each domain of the tool, with which maintains excellent reliability for the tool. Confirmatory factor analysis highlights that the short PSAT retains conceptual distinction across the 8 domains, with CFI scores greater than 0.90, RMSEA scores below 0.6, and SRMR scores less than 0.08. The short CSAT had cronbach’s alpha of 0.84 – 0.92 for each of the domains of the tool, also suggesting excellent reliability of the domains within the measure after dropping two items/domain. Confirmatory factor analysis of the short CSAT meets the same specifications as above, again highlighting conceptual distinction across the domains.

**Conclusion:**

Each tool was able to be shortened to three items per domain while maintaining strong psychometric properties. This results in a tool that takes less time to complete, meeting one of the key calls for pragmatic measures within implementation science. This advances our abilities to measure and test sustainability within implementation science.

**Supplementary Information:**

The online version contains supplementary material available at 10.1186/s43058-024-00656-y.

Contributions to the literature

- This paper presents new short form versions of both the Program Sustainability Assessment Tool and the Clinical Sustainability Assessment Tool
- This project uses sustainability capacity assessments from a variety of programs and practices to understand how to reduce assessment burden and enhance tools for measuring sustainability
- This work advances our access to pragmatic, reliable, and valid tools for implementation science

## Introduction

Evidence-based practices and programs need to be sustained over time to realize their health and social benefits. Many programs and practices never reach sustainment, or the continuation of programs and practices that have been implemented [1–3]. Over the past 10 years, more attention has been paid to measuring and studying sustainability within implementation science. Despite this increased attention, there are still relatively few established measurement tools for assessing sustainability and sustainment [4, 5]. While methodological advances have been made within the area, reviews have still highlighted an need to develop psychometrically tested measures to assess sustainability and sustainment [1]. Additionally, there are opportunities to reduce the burden of previously established sustainability tools by assessing opportunities to shorten the assessment.

Sustainability capacity is the ability to maintain programming and its benefits over time [6]. Our team developed two theoretically informed measures of sustainability capacity, the Program Sustainability Assessment Tool (PSAT) [7, 8], designed to assess sustainability in public health settings, and the Clinical Sustainability Assessment Tool (CSAT) [6, 9], designed to assess sustainability in healthcare settings. These tools were designed to be easy to use by both evaluators and researchers across a variety of contexts and interventions. The PSAT and CSAT have been used in organizational evaluation as well as within public health and implementation science research [10–12]. Both measures had been previously developed with established reliability and validity, and have been widely used across a variety of contexts and settings. Together, the PSAT and CSAT have been completed online over 13,000 times to measure the sustainability capacity of programs and practices across a variety of settings, including public health [10, 13], clinical care [9, 14], behavioral health [15], and education [16]. These measures have aided in distinguishing key determinants and outcomes of sustainability capacity. Other measures of sustainability, sustainment, and implementation science more broadly are still emerging.

As we increase the number of surveys used in implementation research, there is an opportunity to have shorter measures that still provide rigorous measurement and reduce the burden on participants. Pragmatic assessment tools, often described as tools that are usable and brief, are important in implementation science because they ensure consistent measurement of constructs as well as advance implementation practice efforts [17, 18]. A brief measure that retains strong psychometric properties would facilitate greater use of the sustainability capacity measures. Additionally, there has been previous work suggesting the shortening of the CSAT and PSAT specifically would increase usability and uptake in a variety of contexts. To meet this demand, the current study develops and presents brief measures of both clinical and program sustainability capacity. This work is based off the conceptual and measurement work previously developing the PSAT and CSAT and will advance the pragmatic measurement of sustainability.

In this brief report, we: 1) present new shorter forms of the PSAT and CSAT for use by implementation science researchers, and public health and healthcare evaluators; and 2) present psychometric data establishing short and reliable versions of the two assessment instruments.

## Methods

### General design

This study was conducted in two parts, one for the Program Sustainability Assessment Tool (PSAT) and one for the Clinical Sustainability Assessment Tool (CSAT). (See *sustaintool.org* for more information about our sustainability framework and to access these measurement tools.) This psychometrics study takes advantage of previously collected data from applications of the original long forms of the PSAT and CSAT. All participants completed one of our sustainability tools (CSAT or PSAT), along with a brief questionnaire to provide information on the individual demographics, program or practice characteristics, and the participant’s affiliated organization. The PSAT contains eight different domains with five items in each domain for a total of 40 items. Each item is measured on a Likert scale from 1–7, with 7 representing that the concept exists to the fullest extent. The eight domains are: environmental support, funding stability, partnerships, organizational capacity, program evaluation, program adaptation, communications, and strategic planning. The CSAT contains seven different domains with five items in each domain for a total of 35 items. Each item is also measured on a likert scale from 1–7, with 7 being the greatest extent of a concept. The seven domains are: engaged staff and leaders, engaged partners, monitoring and evaluation, outcomes and effectiveness, organizational readiness, implementation and training, and workflow integration.

Individuals did not receive monetary incentives to participate but were provided with reports of their program or clinical sustainability scores. Data were collected from the website where our tool is housed, which has a privacy statement associated with Washington University in St. Louis IRB approval where individuals consent to analysis of de-identified data upon submission. Other data from the CSAT was utilized, which was all collected under IRB approval associated with Washington University in St. Louis. All data collected through other studies has already been reviewed for quality and collected through rigorous survey methods. Data collected through the website was reviewed for quality and validity. All data that was marked as test data, or where the practice listed was ‘test’ were removed prior to analyses. Additionally, data were removed when deemed to be incomplete or test data, which included (1) responses that were the same across all response options or (2) responses where at least one entire domain of data were missing.

### PSAT participants and procedures

Data for the PSAT were pulled from *sustaintool.org,* where an online version of the PSAT is available to be used. This included all individuals who had completed the online tool since it’s online availability from 2014 through 2019. This sample size totaled 5,706 respondents. These individuals were involved in different areas of public health but were assessing sustainability capacity of their program as an individual or group. Individuals were affiliated with the program or practice, but included leadership as well as frontline public health professionals. Participants identified their programs as community (*n* = 3,829), state (*n* = 1,339), national (*n* = 199), international (n = 91), and tribal (*n* = 175). Programs had been in place for varying lengths of time, with the majority of programs in place from 1–3 years (28%). Individuals completed the assessment while considering a program or practice with a primary field focused on topics such as public health, social services, and education.

### CSAT participants and procedures

CSAT data were obtained and combined from multiple sources. This included the website data from sustaintool.org, where individuals completed the CSAT assessment for clinical programs or practices. The online CSAT data were collected from 2018—2022. Additionally, data that had been collected in previous studies of clinical sustainability were included in the total sample for the psychometric analyses. These data included respondents from three different studies of antimicrobial stewardship [19], cancer screening programs, and smoking cessation interventions [14]. These programs were both hospital based and in ambulatory care and included different specialties and organization size. Individuals who were affiliated with the programs and practices, either as leaders or bedside providers, completed these assessments. After combining these data sources, the sample size totaled 501 respondents. Data were most often from practices implemented in academic medical settings (*n* = 219) followed by clinic settings (*n* = 70). Most practices had been in place either 1—2 years (*n* = 123) or 3—5 years (*n* = 129).

### Psychometric analyses

All data management and analyses were conducted in R. For analytic purposes, any responses marked not applicable were considered missing. The goal of these analyses was to determine if a shorter version of each sustainability tool could be constructed using just three items from the original five items per subscale. Three items were chosen so that the resulting instrument would be noticeably shorter than the original long form, but still maintaining the ability to measure and demonstrate subscale reliability and multidomain measurement structures (where 3 items per scale are generally required for confirmatory factor analyses) [20].

First, Cronbach’s alpha was calculated to assess the internal consistency of the original five item subscales. Candidate items to be dropped were the items with the lowest item-total correlation with the relevant subscale. Following this, the team met for substantive review to compare options and select the items to drop to create the short versions. The team selected two items to drop from each subscale, informed by the psychometric analyses. Internal consistency was recalculated for the new three-item subscales. Confirmatory factor analysis was also conducted to assess the measurement structure of the original long-form and new short-form versions. The research team met to review and examine factors, with attention paid to understanding differentiation, utility, and face validity of the new proposed subscale domains.

## Results

### Program sustainability assessment tool

#### PSAT reliabilities

Table 1 presents the subscale reliabilities (internal consistency) for the PSAT for the original published version and for the tool with 3 items/domain. Two items were dropped from each of the original measures through a group review of internal consistency and theoretical importance, dropping the two lowest performing items in each domain while retaining conceptually important items. Cronbach’s alpha decreased slightly for each domain, which is to be expected for shorter subscales. However, all domains retained an alpha above 0.80, suggesting excellent reliability for each of the eight subscales.

**Table 1:** Subscale Internal consistencies of original long-form PSAT and new short-form version

*Subdomain*	*Original items*	*Original Cronbach’s Alpha*	*New items*	*Short Cronbach’s Alpha*
Environmental support	5	.851	3	.825
Funding stability	5	.879	3	.871
Partnerships	5	.921	3	.896
Organizational capacity	5	.889	3	.824
Program evaluation	5	.911	3	.897
Program adaptation	5	.924	3	.908
Communications	5	.942	3	.912
Strategic planning	5	.894	3	.825

#### PSAT measurement structure

Confirmatory factor analysis was used to assess the structure of the long and short PSAT with this data set. The originally published model was used as a baseline comparison. This model includes all items contained in the 8 subscales with a pilot sample of 592 participants. After collecting additional responses, Table 2 shows the fit of the additional confirmatory factor analyses. The CFA results highlight excellent fit of the eight PSAT domains both in the long and short versions. Factor loadings for each retained item can be found in Supplemental File C. The CFI scores greater than 0.90, RMSEA scores below 0.6, and SRMR scores less than 0.08 suggest that both the long and short version of the PSAT have excellent fit [21]. This suggests that the short version the PSAT retains conceptual distinction across the 8 domains, while still measuring an interrelated concept of program sustainability.

**Table 2:** Confirmatory factor analysis results assessing the eight subscale domain measurement structure of original long-form PSAT and new short-form version

*Version*	*N*	*Chi/df*	*CFI*	*RMSEA*	*SRMR*
Original published (5 items)	592	3.6	.890	.066	.063
Updated long version (5 items)	5706	17.6	.902	.054	.054
Short version (3 items)	5706	9.9	.969	.040	.036

#### PSAT short-form tool

The PSAT shortened tool (Supplemental File A) retains all eight domains of the original measure, with confirmatory factor analysis supporting the domain structure as originally published. The eight domains are: Environmental Support, Funding Stability, Partnerships, Organizational Capacity, Program Evaluation, Program Adaptation, Communications, and Strategic Planning. This results in a 24-item PSAT measure, which is 40% shorter than the original long version. All items are still measured on a 7-point Likert Scale, with scores calculated as an average of each domain and overall.

### Clinical sustainability assessment tool

#### CSAT reliabilities

Cronbach’s alpha was calculated for each of the original domains. Low performing items within each domain were reviewed by our team and items were selected to drop. Items that were retained were selected by reviewing both psychometric performances along with conceptual importance. Two items within each domain were selected to drop, resulting in three questions within each of the seven domains. Table 3 presents the Cronbach’s alpha for the original tool domains along with the shortened tool. After dropping items, each domain had a Cronbach’s alpha of 0.80 or above, highlighting excellent internal consistency of each of the seven domains.

**Table 3:** Subscale internal consistencies of original long-form CSAT and new short-form version

*Subdomain*	*Original items*	*Original Cronbach’s Alpha*	*New items*	*Short Cronbach’s Alpha*
Engaged staff	5	.899	3	.869
Engaged partners	5	.858	3	.842
Organizational readiness	5	.897	3	.862
Workflow integration	5	.908	3	.894
Implementation & training	5	.915	3	.870
Monitoring & evaluation	5	.940	3	.886
Outcomes & effectiveness	5	.917	3	.924

#### CSAT measurement structure

After reviewing internal consistency and selecting three items, we conducted Confirmatory Factor Analysis to assess the structure of both the original measure and new shortened measure with this data set (Table 4). Both the original measure and the shortened measure had excellent fit, with values improved over the originally published CSAT measure. The factor loadings for each item can be found in Supplemental File C. The CFI scores greater than 0.90, RMSEA scores below 0.6, and SRMR scores less than 0.08 suggest that the short version of the CSAT has excellent fit. This suggests that the seven-factor structure remains appropriate, both for the original measure and the shortened version.

**Table 4:** Confirmatory factor analysis results assessing the seven subscale domain measurement structure of original long-form CSAT and new short-form version

*Version*	*N*	*Chi/df*	*CFI*	*RMSEA*	*SRMR*
Original published (5 items)	120	1.84	.810	.084	.075
Updated long version (5 items)	501	2.52	.912	.055	.048
Short version (3 items)	501	2.56	.946	.056	.045

#### CSAT short-form tool

The short CSAT (Supplemental File B) is comprised of seven domains, with three items in each domain. The seven domains retain their original names: Engaged Staff, Engaged Partners, Organizational Readiness, Workflow Integration, Implementation and Training, Monitoring and Evaluation, and Outcomes and Effectiveness. The measure is 21 questions long, which is 40% shorter than the original long version. Each item is measured on a 7-point Likert scale. Scores are an average of each domain along with an overall score average.

## Discussion

This study developed two brief measures of sustainability capacity – a short PSAT and short CSAT measure – to assess sustainability capacity for programs and practices. After using the analyses to select items to drop, each measure has 3 items per domain. The short PSAT has 8 domains, for a total of 24 items. The short CSAT measure has 7 domains, for 21 items. We were able to reduce the number of items of both measures while maintaining strong psychometric properties, and, in doing so, reduce the burden of assessment and facilitate measurement of sustainability capacity in research, evaluation, and practice setting [5, 17, 18, 22]. Especially for research and evaluation in clinical settings were clinician time is extremely limited, shorter versions of the tool will help support more effective participation among clinicians. This work will aid in the development of interventions and strategies that respond to identified gaps based on these measures, which will serve to further advance sustainability of evidence informed programs and practices [1].

The PSAT and CSAT have been utilized across multiple research and practice settings, although the field has broadly recognized the need for more pragmatic research tools. Many studies, especially within implementation science, have a large number of surveys that results in high burden. Further, evaluation and quality improvement programs are aware of the burden that can result from this work, increasing the importance of easy-to-use tools. This is especially true in under resourced settings, especially globally [9]. Previous work using and evaluating the PSAT and CSAT have commented on their pragmatic qualitites [5, 16]. Within implementation science, the PAPERS scale has been adopted as a way to assess the pragmatic nature of measures and tools. While the PAPERS scale has a cutoff of 10 items for a short tool [22], reducing the PSAT and CSAT still reduces the burden of assessment. Our previous work has commented on the length of the assessment and suggested that shortening the tool would result in higher usability [11]. Other studies have used the PSAT or CSAT in conjunction with other measures, where a shorter scale will improve overall burden while maintaining high rigor.

The introduction of the short version of these tools is not intended to replace the longer version. Recommendations for choosing to use the short version include concerns about participant burden and when used with the inclusion of other measures. However, some scenarios might call for the use of the original, longer measures. There are items in the longer version that might be important for practices and programs. Further, there are scenarios when individuals might want to understand more comprehensive and nuanced understanding of domains, which would call for using all items in the original measure. Especially in cross sectional designs, which only allows for a single measurement, the original, more comprehensive tool remains a useful assessment of sustainability capacity. Items that were removed during shortening were in attempts to shorten the tool, but the tool was not shortened due to the original containing more items than necessary. Items were dropped by reviewing both the content as an expert team alongside the psychometric analyses, in the same procedure that the original tool was developed. All versions of the measures can be found online at sustaintool.org, and the short versions are made available through supplementary materials in this manuscript. Of note, during this process, the word stakeholder was also replaced with other language throughout both the PSAT and CSAT. This terminology was updated to increase the cultural sensitivity and inclusivity of the tool. An expert team reviewed all the instances where the term was changed to ensure that interpretation of the items would remain consistent. In future work, we plan to collect data using this new language for future psychometric analyses.

While the analyses suggest that the short CSAT and PSAT are reliable measures of sustainability capacity, they were used in data collection for other purposes. However, there were large amounts of data available, creating a data set that we believe to be representative of a broad spectrum of interventions, practitioners, and settings. All data were collected using the longer versions of the tools, but future work should test data collected on both versions of these measures with a new sample. However, these results are promising, and future work can explore questions with new data generation.

## Conclusion

This work responds to calls within implementation science to for pragmatic measures [17, 18, 22], which include a criterion for shorter tools to reduce burden of assessment. This manuscript contributes shorter measures for sustainability capacity that are both theoretically driven and empirically tested. The short PSAT and short CSAT are reliable, precise, and pragmatic measures to further develop our understanding of sustainability of evidence informed programs and practices.

## Supplementary Information

Supplementary Material 1.

Supplementary Material 2.

Supplementary Material 3.

## Data Availability

The datasets used and/or analyzed during the current study are available from the corresponding author on reasonable request.

## Abbreviations

PSAT: Program sustainability assessment tool
CSAT: Clinical sustainability assessment tool
CFI: Comparative fit index
RMSEA: Root mean square error of approximation
SRMR: Standardized Root Mean Residual
CFA: Confirmatory factor analysis
