# Supplementary material for: Lowering the burden: Shorter versions of the Program Sustainability Assessment Tool (PSAT) and Clinical Sustainability Assessment Tool (CSAT)
Source: Implement Sci Commun. 2024 Oct 10;5:113. doi: 10.1186/s43058-024-00656-y (PMC11468075; doi:10.1186/s43058-024-00656-y)
Supplement: Supplementary file 1 — Supplementary Material 1. [file 43058_2024_656_MOESM1_ESM.docx]

**Supplemental Material A. PSAT – Short version**

| **PSAT** | **Short PSAT** |
| --- | --- |
| **Environmental Support:** Having a supportive internal and external climate for your program | **Environmental Support:** Having a supportive internal and external climate for your program |
| 1. Champions exist who strongly support the program. | 1. Champions exist who strongly support the program. |
| 1. The program has strong champions with the ability to garner resources. | 1. The program has strong champions with the ability to garner resources. |
| 1. The program has leadership support from within the larger organization. |  |
| 1. The program has leadership support from outside of the organization. | 1. The program has leadership support from outside of the organization. |
| 1. The program has strong public support. |  |
| **Funding Stability**: Establishing a consistent financial base for your program | **Funding Stability**: Establishing a consistent financial base for your program |
| 1. The program exists in a supportive state economic climate. |  |
| 1. The program implements policies to help ensure sustained funding. |  |
| 1. The program is funded through a variety of sources. | 1. The program is funded through a variety of sources. |
| 1. The program has a combination of stable and flexible funding. | 1. The program has a combination of stable and flexible funding. |
| 1. The program has sustained funding. | 1. The program has sustained funding. |
| **Partnerships:** Cultivating connections between your program and its partners | **Partnerships:** Cultivating connections between your program and its partners |
| 1. Diverse community organizations are invested in the success of the program. |  |
| 1. The program communicates with community leaders. | 1. The program communicates with community leaders. |
| 1. Community leaders are involved with the program. | 1. Community leaders are involved with the program. |
| 1. Community members are passionately committed to the program. |  |
| 1. The community is engaged in the development of program goals. | 1. The community is engaged in the development of program goals. |
| **Organizational Capacity:** Having the internal support and resources needed to effectively manage your program and its activities | **Organizational Capacity:** Having the internal support and resources needed to effectively manage your program and its activities |
| 1. The program is well integrated into the operations of the organization. | 1. The program is well integrated into the operations of the organization. |
| 1. Organizational systems are in place to support the various program needs. | 1. Organizational systems are in place to support the various program needs. |
| 1. Leadership effectively articulates the vision of the program to external partners. |  |
| 1. Leadership efficiently manages staff and other resources. |  |
| 1. The program has adequate staff to complete the program’s goals. | 1. The program has adequate staff to complete the program’s goals. |
| **Program Evaluation:** Assessing your program to inform planning and document results | **Program Evaluation:** Assessing your program to inform planning and document results |
| 1. The program has the capacity for quality program evaluation. |  |
| 1. The program reports short term and intermediate outcomes. | 1. The program reports short term and intermediate outcomes. |
| 1. Evaluation results inform program planning and implementation. | 1. Evaluation results inform program planning and implementation. |
| 1. Program evaluation results are used to demonstrate successes to funders and other interested parties. | 1. Program evaluation results are used to demonstrate successes to funders and other interested parties. |
| 1. The program provides strong evidence to the public that the program works. |  |
| **Program Adaptation:** Taking actions that adapt your program to ensure its ongoing effectiveness | **Program Adaptation:** Taking actions that adapt your program to ensure its ongoing effectiveness |
| 1. The program periodically reviews the evidence base. |  |
| 1. The program adapts strategies as needed. | 1. The program adapts strategies as needed. |
| 1. The program adapts to new science. | 1. The program adapts to new science. |
| 1. The program proactively adapts to changes in the environment. | 1. The program proactively adapts to changes in the environment. |
| 1. The program makes decisions about which components are ineffective and should not continue. |  |
| **Communications:** Strategic communication with partners and the general public about your program | **Communications:** Strategic communication with partners and the general public about your program |
| 1. The program has communication strategies to secure and maintain public support. |  |
| 1. Program staff communicate the need for the program to the public. | 1. Program staff communicate the need for the program to the public. |
| 1. The program is marketed in a way that generates interest. |  |
| 1. The program increases community awareness of the issue. | 1. The program increases community awareness of the issue. |
| 1. The program demonstrates its value to the public. | 1. The program demonstrates its value to the public. |
| **Strategic Planning:** Using processes that guide your program’s direction, goals, and strategies | **Strategic Planning:** Using processes that guide your program’s direction, goals, and strategies |
| 1. The program plans for future resource needs. | 1. The program plans for future resource needs. |
| 1. The program has a long-term financial plan. |  |
| 1. The program has a sustainability plan. | 1. The program has a sustainability plan. |
| 1. The program’s goals are understood by all interested parties. |  |
| 1. The program clearly outlines roles and responsibilities for all partners. | 1. The program clearly outlines roles and responsibilities for all partners. |
