# Supplementary material for: Lowering the burden: Shorter versions of the Program Sustainability Assessment Tool (PSAT) and Clinical Sustainability Assessment Tool (CSAT)
Source: Implement Sci Commun. 2024 Oct 10;5:113. doi: 10.1186/s43058-024-00656-y (PMC11468075; doi:10.1186/s43058-024-00656-y)
Supplement: Supplementary file 2 — Supplementary Material 2. [file 43058_2024_656_MOESM2_ESM.docx]

**Supplemental Material B. CSAT – Short version**

| **CSAT** | **Short CSAT** |
| --- | --- |
| **Engaged Staff & Leadership:** Having supportive frontline staff and management within the organization | **Engaged Staff & Leadership:** Having supportive frontline staff and management within the organization |
| 1. The practice engages leadership and staff throughout the process. |  |
| 1. Clinical champions of the practice are recognized and respected. |  |
| 1. The practice has engaged, ongoing champions. | 1. The practice has engaged, ongoing champions. |
| 1. The practice has a leadership team made of multiprofessional partnership. | 1. The practice has a leadership team made of multiprofessional partnership. |
| 1. The practice has team-based collaboration and infrastructure. | 1. The practice has team-based collaboration and infrastructure. |
| **Engaged Partners:** Having external support and engagement for the practice. | **Engaged Partners:** Having external support and engagement for the practice. |
| 1. The practice engages the patient and family members as partners. |  |
| 1. There is respect for all partners involved in the practice. | 1. There is respect for all partners involved in the practice. |
| 1. The practice is valued by a diverse set of interested parties. | 1. The practice is valued by a diverse set of interested parties. |
| 1. The practice engages other medical teams and community partnerships as appropriate. | 1. The practice engages other medical teams and community partnerships as appropriate. |
| 1. The practice team has the ability to respond to feedback about the practice. |  |
| **Organizational Readiness:** Having the internal support and resources needed to effectively manage the practice | **Organizational Readiness:** Having the internal support and resources needed to effectively manage the practice |
| 1. Organizational systems are in place to support the various practice needs. | 1. Organizational systems are in place to support the various practice needs. |
| 1. The practice fits in well with the culture of the team. |  |
| 1. The practice has feasible and sufficient resources (e.g., time, space, funding) to achieve its goals. | 1. The practice has feasible and sufficient resources (e.g., time, space, funding) to achieve its goals. |
| 1. The practice has adequate staff to achieve its goals. | 1. The practice has adequate staff to achieve its goals. |
| 1. The practice is well integrated into the operations of the organization. |  |
| **Workflow Integration:** Designing the practice to fit into existing practices and technologies | **Workflow Integration:** Designing the practice to fit into existing practices and technologies |
| 1. The practice is built into the clinical workflow. | 1. The practice is built into the clinical workflow. |
| 1. The practice is easy for clinicians to use. | 1. The practice is easy for clinicians to use. |
| 1. The practice integrates well with established clinical practices. | 1. The practice integrates well with established clinical practices. |
| 1. The practice aligns well with other clinical systems (e.g., EMR). |  |
| 1. The practice is designed to be used consistently. |  |
| **Implementation & Training:** Using processes that guide the direction, goals and strategies of the practice | **Implementation & Training:** Using processes that guide the direction, goals and strategies of the practice |
| 1. The practice clearly outlines roles and responsibilities for all staff. |  |
| 1. The reason for the practice is clearly communicated to and understood by all staff. | 1. The reason for the practice is clearly communicated to and understood by all staff. |
| 1. Staff receive ongoing coaching, feedback, and training. | 1. Staff receive ongoing coaching, feedback, and training. |
| 1. Practice implementation is guided by feedback. |  |
| 1. The practice has ongoing education across professions. | 1. The practice has ongoing education across professions. |
| **Monitoring & Evaluation:** Assessing the practice to inform planning and document results | **Monitoring & Evaluation:** Assessing the practice to inform planning and document results |
| 1. The practice has measurable process components, outcomes, and metrics. | 1. The practice has measurable process components, outcomes, and metrics. |
| 1. Evaluation and monitoring of the practice are reviewed on a consistent basis. | 1. Evaluation and monitoring of the practice are reviewed on a consistent basis. |
| 1. The practice has clear documentation to guide process and outcome evaluation. |  |
| 1. Practice monitoring, evaluation, and outcomes data are routinely reported to the clinical care team. | 1. Practice monitoring, evaluation, and outcomes data are routinely reported to the clinical care team. |
| 1. The practice process components, outcomes, and metrics are easily assessed and audited. |  |
| **Outcomes & Effectiveness:** Understanding and measuring practice outcomes and impact | **Outcomes & Effectiveness:** Understanding and measuring practice outcomes and impact |
| 1. The practice has evidence of beneficial outcomes. | 1. The practice has evidence of beneficial outcomes. |
| 1. The practice is associated with improvement in patient outcomes that are clinically meaningful. | 1. The practice is associated with improvement in patient outcomes that are clinically meaningful. |
| 1. The practice is clearly linked to positive health or clinical outcomes. | 1. The practice is clearly linked to positive health or clinical outcomes. |
| 1. The practice is cost-effective. |  |
| 1. The practice has clear advantages over alternatives. |  |
