# Supplementary material for: Lowering the burden: Shorter versions of the Program Sustainability Assessment Tool (PSAT) and Clinical Sustainability Assessment Tool (CSAT)
Source: Implement Sci Commun. 2024 Oct 10;5:113. doi: 10.1186/s43058-024-00656-y (PMC11468075; doi:10.1186/s43058-024-00656-y)
Supplement: Supplementary file 3 — Supplementary Material 3. [file 43058_2024_656_MOESM3_ESM.docx]

Supplemental File C.

Final short PSAT (Program Sustainability Assessment Tool) confirmatory factor analysis model fit, with standardized item loadings for each PSAT domain.


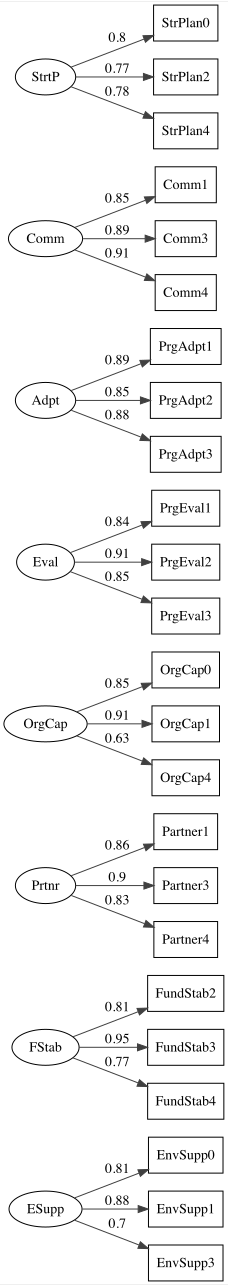


Final short PSAT (Program Sustainability Assessment Tool) confirmatory factor analysis model fit table of factor correlation coefficient

|  | Environmental Support | Funding Stability | Partnerships | Organizational Capacity | Program Evaluation | Program Adaptation | Communications | Strategic Planning |
| --- | --- | --- | --- | --- | --- | --- | --- | --- |
| Environmental Support | 1.0 |  |  |  |  |  |  |  |
| Funding Stability | 0.48 | 1.0 |  |  |  |  |  |  |
| Partnerships | 0.58 | 0.45 | 1.0 |  |  |  |  |  |
| Organizational Capacity | 0.60 | 0.52 | 0.52 | 1.0 |  |  |  |  |
| Program Evaluation | 0.52 | 0.39 | 0.52 | 0.63 | 1.0 |  |  |  |
| Program Adaptation | 0.56 | 0.36 | 0.50 | 0.61 | 0.73 | 1.0 |  |  |
| Communications | 0.53 | 0.46 | 0.69 | 0.57 | 0.58 | 0.57 | 1.0 |  |
| Strategic Planning | 0.65 | 0.64 | 0.62 | 0.72 | 0.72 | 0.70 | 0.73 | 1.0 |

Final short CSAT (Clinical Sustainability Assessment Tool) confirmatory factor analysis model fit, with standardized item loadings for each CSAT domain.


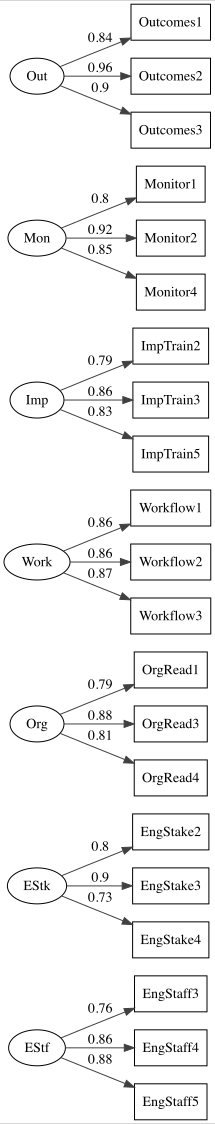


Final short CSAT (Clinical Sustainability Assessment Tool) confirmatory factor analysis model fit table of factor correlation coefficient

|  | Engaged Staff | Engaged Partners | Organization Readiness | Workflow Integration | Implementation and Training | Monitoring and Evaluation | Outcomes and Effectiveness |
| --- | --- | --- | --- | --- | --- | --- | --- |
| Engaged Staff | 1.0 |  |  |  |  |  |  |
| Engaged Partners | 0.76 | 1.0 |  |  |  |  |  |
| Organization Readiness | 0.59 | 0.61 | 1.0 |  |  |  |  |
| Workflow Integration | 0.63 | 0.63 | 0.73 | 1.0 |  |  |  |
| Implementation and Training | 0.64 | 0.64 | 0.65 | 0.74 | 1.0 |  |  |
| Monitoring and Evaluation | 0.57 | 0.52 | 0.62 | 0.64 | 0.78 | 1.0 |  |
| Outcomes and Effectiveness | 0.49 | 0.49 | 0.46 | 0.56 | 0.56 | 0.56 | 1.0 |
